# Exploring the Influence of Synthesis Parameters on the Optical Properties for Various CeO_2_ NPs

**DOI:** 10.3390/nano12091402

**Published:** 2022-04-19

**Authors:** Andreea L. Chibac-Scutaru, Viorica Podasca, Ioan A. Dascalu, Violeta Melinte

**Affiliations:** 1 Polyaddition and Photochemistry Department, Petru Poni Institute of Macromolecular Chemistry, 41A Grigore Ghica Voda Alley, 700487 Iasi, Romania; andreea.chibac@icmpp.ro (A.L.C.-S.); podasca.viorica@icmpp.ro (V.P.); 2 Centre of Advanced Research in Bionanoconjugates and Biopolymers (IntelCentru), Petru Poni Institute of Macromolecular Chemistry, 41A GrigoreGhicaVoda Alley, 700487 Iasi, Romania; idascalu@icmpp.ro

**Keywords:** CeO_2_ nanoparticles, pH variation, Co-doping, optical properties, band gap tuning

## Abstract

Cerium oxide (CeO_2_) nanoparticles were synthesized with a chemical precipitation method in different experimental conditions using cerium nitrate hexahydrate (Ce(NO_3_)_3_·6H_2_O) as a precursor, modifying the solution pH, the reaction time, and Co atoms as dopants, in order to tune the band gap energy values of the prepared samples. The physical characteristics of the synthesized ceria nanoparticles were evaluated by Fourier transform infrared (FT-IR) spectroscopy, X-ray diffraction (XRD), scanning electron microscopy (SEM), transmission electron microscopy (TEM), UV–Vis analyses and photoluminescence measurements. XRD data revealed a pure cubic fluorite structure of CeO_2_ NPs, the estimation of crystallite sizes by Scherrer’s formula indicates the formation of crystals with dimensions between 11.24 and 21.65 nm. All samples contain nearly spherical CeO_2_ nanoparticles, as well as cubic, rhomboidal, triangular, or polyhedral nanoparticles that can be identified by TEM images. The optical investigation of CeO_2_ samples revealed that the band gap energy values are between 3.18 eV and 2.85 eV, and, after doping with Co atoms, the *E_g_* of samples decreased to about 2.0 eV. In this study, we managed to obtain CeO_2_ NPs with *E_g_* under 3.0 eV by only modifying the synthesis parameters. In addition, by doping with Co ions, the band gap energy value was lowered to 2.0 eV. This aspect leads to promising results that provide an encouraging approach for future photocatalytic investigations.

## 1. Introduction

The synthesis of ceria nanoparticles (CeO_2_ NPs) of various sizes and shapes was thoroughly investigated in recent research due to their promising chemical and physical properties and their potential applications as catalysts [1,2,3,4], ultraviolet absorbers [5], oxygen sensors [6], or storage systems [7,8]. The physicochemical features of ceria nanoparticles are closely related to their crystalline structure, the reason for this is that, over time, different techniques have been developed to synthesize CeO_2_ nanoparticles, such as hydrothermal technique [9,10], sol–gel method [11,12], precipitation [13,14], solvothermal [15], microemulsion [16], microwave [17] methods, or their combination [18,19]. Depending on the chosen synthesis method, the size, shape, and crystallinity of the nanoparticles are modified as a function of the synthesis parameters, namely the reactant concentration, reaction time and temperature, solution pH, and the use of surfactants [10,20,21,22,23,24,25]. Generally, the synthetic methods are preferred because they provides convenient attributes, such as short preparation time, low reaction temperature and low cost, high purity and homogeneity of the resulting particles, and well-crystallized products with predictable size and morphology [26,27,28,29].

If CeO_2_ nanoparticles are intended for photocatalytic applications, an extreme importance is credited to the optical properties and especially to the band gap value, which dictates the potential use of nanoparticles in the UV or visible field. It is well documented that CeO_2_ NPs can display a wide band gap energy of 2.6 to 3.4 eV [1,30,31], which can be modulated by the preparation method; moreover, it was proved that it depends on the presence of oxygen vacancies and Ce^3+^ ions [9,32,33].

Another option to improve the photocatalytic activity of CeO_2_ in terms of band gap decrease refers to rare earth or transition metal doping [34,35], which could induce the formation of lattice defects able to selectively tune the electronic structure of CeO_2_ by a better separation of h^+^/e^−^ pairs, leading to the enhanced absorption of visible light [35,36]. The improvement of optical and catalytic properties induced by metal ion doping is very sensitive to their structural and electronic properties, thus there are many factors such as the nature of the dopant, synthesis route, size, morphology, band gap, and the type of defects responsible for tuning the photonic characteristics [35,36,37].

In this context, the paper focuses on the synthesis of CeO_2_ NPs in diverse preparative conditions (pH, time, co-reagent variation), followed by a methodical characterization, in order to evaluate their structural, morphological, and optical attributes, with an emphasis on the most accurate correlation of structural, optical, and morphological results. Our attention was especially focused on the modulation of the band gap energy of CeO_2_ NPs in order to facilitate a better harvesting of light, especially visible light, in various photocatalytic processes. Thus, in an attempt to enlarge the photocatalytic activity of the prepared nanoparticles, we succeeded to demonstrate that CeO_2_ NPs with the desired *E_g_* values (under 3.0 eV) can be easily obtained only by a convenient modification of the synthesis parameters and that, in addition, by doping with Co ions, the band gap energy value was lowered to 2.0 eV. The performed investigations will allow us to select the optimal synthesis parameters for the preparation by a facile method of CeO_2_ NPs, promising for photocatalytic application under sunlight, with predictable values of *E_g_*, sizes and a slow recombination rate of charge carriers.

## 2. Materials and Methods

### 2.1. Materials and Reagents

Cerium nitrate hexahydrate (Ce(NO_3_)_3_·6H_2_O), cobalt nitrate hexahydrate (Co(NO_3_)_2_·6H_2_O), ammonia solution 25%, NaOH, terephthalic acid, and ethanol 96% were purchased from Sigma–Aldrich Co. (Taufkirchen, Germany) and used as received. Deionized water was used in the experiments and in the preparation of the solutions.

### 2.2. Preparation of CeO_2_ Nanoparticles

#### 2.2.1. Synthesis of CeO_2_ Nanoparticles V1, V2 and V3

The first series of CeO_2_ NPs discussed in this study was synthesized according to a procedure reported in the literature by Podasca et al. for the synthesis of ZnO nanoparticles [38]. Thus, the synthesis steps for the preparation of V1 NPs were as follows: in a 500 mL round bottomed flask fitted with a mechanical stirrer, 6 g (14 mmol) cerium nitrate hexahydrate was dissolved in 350 mL deionized water. Ammonia solution (25%) was then added into the cerium solution under magnetic stirring until pH = 8, and the reaction mixture was left stirring for 3h at 85 °C. The precipitate was centrifuged, washed with deionized water, and dried at room temperature for 1 day, and then the dried sample was calcined at 600 °C for 3 h. In the preparation of V2 NPs, the solution was adjusted to pH 10 using ammonia (25%); the rest of the synthesis protocol remaining unchanged. For the preparation of V3 NPs, the reaction mixture at pH = 10 was heated at 85 °C for 72 h.

#### 2.2.2. Synthesis of CeO_2_ NPs by Version 2 (V4)

This synthesis was realized by using a similar method as Dong and co. [39], though with a few modifications. In a 100 mL bottomed flask fitted with a mechanical stirrer, 1.74 g (4 mmol) cerium nitrate hexahydrate was dissolved in 40 mL deionized water and, under vigorous stirring, 0.3 M solution of sodium hydroxide was dropwise added up to pH 12. The solution was heated at 100 °C and was maintained at this temperature for 3 h in order to allow the formation of CeO_2_ particles. When cooled down to room temperature, the precipitate was centrifuged, washed with deionized water, dried at room temperature for 1 day, and the sample was calcined at 600 °C for 3 h.

#### 2.2.3. Synthesis of CeO_2_ NPs by Version 3 (V5, V6)

This synthesis protocol was adapted from Li et al. [40]. Thus, 1.74 g (4 mmol) cerium nitrate hexahydrate and 0.66 g (4 mmol) terephthalic acid were dissolved in a 1:1 ethanol-water solution (200 mL, *v/v* = 1:1) under vigorous stirring at room temperature. Then, the stirring of the solution was continued for 3 h at 90 °C until the formation of a white precipitate was noticed. Further, the temperature of the reaction mixture was reduced to 24 °C, the formed precipitate was collected by centrifugation, and, for an additional purification, was washed several times with water and ethanol, followed by overnight drying in an oven at 60 °C. The resulting product was calcined at 600 °C in air atmosphere for 3 h and the V5 sample was achieved. For the synthesis of V6 NPs, the same protocol was followed, except that the stirring of the reaction mixture was prolonged by up to 72 h at a temperature of 90 °C.

### 2.3. Preparation of Co-Doped CeO_2_ Nanoparticles (V2-Co and V5-Co)

For the synthesis of Co-doped CeO_2_ NPs, V2 and V5 samples were selected. The same synthesis pathway as for the undoped samples was pursued, except that, initially, the reaction mixtures contain in each case a 9:1 molar ratio of Ce(NO_3_)_3_·6H_2_O: Co(NO_3_)_2_·6H_2_O. For an easier identification of the samples, the preparation conditions are summarized in Table 1.

### 2.4. Characterization

Bruker Vertex 70 FTIR spectrophotometer (BRUKER, Karlsruhe, Germany) was used to register the Fourier transform infrared (FTIR) spectra on KBr pellets in the spectral domain 400–4000 cm^−1^. For the evaluation of the crystalline structure of CeO_2_ NPs, a Rigaku Miniflex 600 X-ray diffractometer (Tokyo, Japan) was employed. The diffractometer used Cu Kα radiation (*λ* = 1.5406 Å) and the measurements were performed in the range 2*θ* = 20.0°–90.0°, in steps of 0.01° and a recording rate of 1°/min. To calculate the average crystallite size of CeO_2_ NPs from the XRD diffractograms, the Scherrer equation was applied. The size and morphology of CeO_2_ NPs were investigated on a HITACHI T7700 transmission electron microscope (TEM) (Tokyo, Japan) at an accelerating voltage of 120 kV. For the analysis, CeO_2_ NPs were deposited from an ethanol solution on a carbon-coated copper grid. The agglomeration of ceria particles on the copper grid can be a consequence of the drying process. The surface morphology and energy-dispersive X-ray spectroscopy (EDX) investigations were carried out on a Verios G4 UC scanning electron microscope (Thermo Fisher Scientific, Brno, Czech Republic) coupled with an energy dispersive spectrometer (EDS, EDAX Octane Elite). For high-quality SEM images, the investigated NPs were sputter coated with a thin layer of platinum of 6 nm. For the measurement of the optical properties of the samples, CeO_2_ NPs (pristine and Co-doped) were rigorously dispersed in ethanol (1 mg/10 mL) by sonication for 30 min. Further, the UV–Vis absorption spectra were recorded for each solution in the wavelength domain 200–1000 nm using a Perkin Elmer Lambda 2 UV–Vis spectrophotometer (Perkin Elmer Inc., Wellesley, MA, USA). The reflectance spectra of the pristine and Co-doped CeO_2_ NPs were also measured in the wavelength domain 300–1000 nm. The photoluminescence spectra at different excitation wavelengths (270 and 325 nm) were registered on an RF-6000 Shimadzu spectrofluorometer (SHIMADZU, Korneuburg, Austria).

## 3. Results and Discussion

### 3.1. Structural and Morphological Characterization

**Figure 1:**
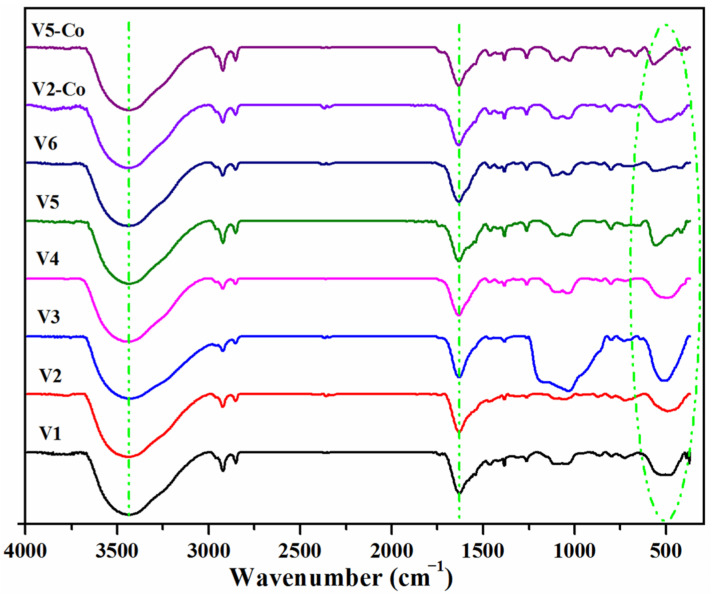
FTIR spectra of CeO_2_ nanoparticles prepared using various synthesis methods.

The FTIR spectra of the prepared samples in the range of 4000–400 cm^−1^ are illustrated in Figure 1. In all spectra, a large band with a maximum at around 3435 cm^−1^, accompanied by another band centered at ~1632 cm^−1^, are visible, and may be attributed respectively to the stretching and bending mode of the physisorbed water molecules in the samples. The wide absorption band at around 400–600 cm^−1^ can be attributed to Ce–O stretching vibrations, and it confirms the formation of CeO_2_ particles. The other absorption bands present in all IR spectra might be assigned to various absorbing species (carbonate-type species such as atmospheric CO_2_, for example, which could interact with the cerium cations at 1000–1200 cm^−1^ or the band at 1385 cm^−1^ given by the stretch of N–O linkages coming from nitrate precursors) [23,41]. Although the samples were calcined at 600 °C after preparation, absorption bands located in the 2800–3000 cm^−1^ region are visible in all FTIR spectra and can be assigned to C–H hydrocarbon stretching and are a measure of ceria reactivity at the surface level [23] or can be associated to the presence of some residual organic molecules [42].

**Figure 2:**
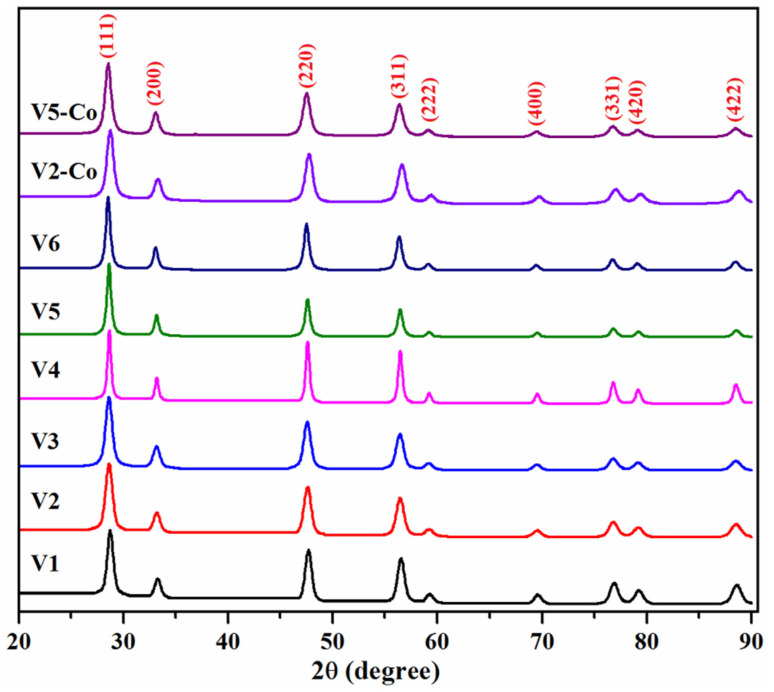
XRD patterns of CeO_2_/Co-doped CeO_2_ nanoparticles.

The phase purity of CeO_2_ NPs was evaluated by XRD, and the recorded patterns were illustrated in Figure 2. As can be seen in the figure, the diffraction peaks observed in the 2*θ* range from 20° to 90° (2*θ* = 28.63, 33.17, 47.60, 56.44, 59.22, 69.50, 76.82, 79.18, 88.55) can be associated with (111), (200), (220), (311), (222), (400), (331), (420), and (422) planes, respectively, and share a close resemblance to literature data for CeO_2_ NPs (JCPDS 43-1002) corresponding to the pure cubic fluorite structure of CeO_2_. Moreover, in the doped samples, the absence of any extra diffraction peaks attributable to cobalt oxides suggests the isomorphic replacement of cerium by cobalt cations in the cubic lattice or their high dispersion in the ceria crystals [43].

The estimation of the crystallite sizes of the samples was performed using Scherrer’s formula, *d* = K*λ*/*β*cos*θ*, where *d* is the crystallite size, K is the so-called shape factor, which usually takes a value of about 0.9, *λ* is the X-ray wavelength of the radiation source (1.5406 Å), *β* is the full-width at half-maximum (FWHM) of the diffraction peak, and *θ* is the Bragg angle. The crystal size varies between 11.24 and 21.65 nm (Table 2). The nanoparticles with smallest crystallite sizes (~11–12 nm) were obtained through the reactions achieved at pH 8 and 10 (V1–V3 samples), while the pH variation at more extreme values (4 or 12) leads to a doubling of the crystallite dimension (~20 nm) of the nanoparticles (V4–V6 samples). If CeO_2_ NPs are intended to be use as photocatalysts, the ones with a smaller crystallite size are preferred, since the charge migration pathway to the surface is reduced compared to the ones with larger dimensions, thus resulting in a decrease in the recombination rate of the charge carriers [44,45]. Moreover, it can be observed that the increase in the reaction time (in the case of V3 and V6 samples) does not cause a significant change in the size of the crystallites. The inclusion of Co atoms in the V2 sample does not notably influence the crystallites’ growth, while, for sample V5-Co, a decrease in the crystallite size was observed, a finding that could be attributed to the replacement of Ce ions with Co-dopant ions, as previously reported by Ranjith et al. [46].

**Figure 3:**
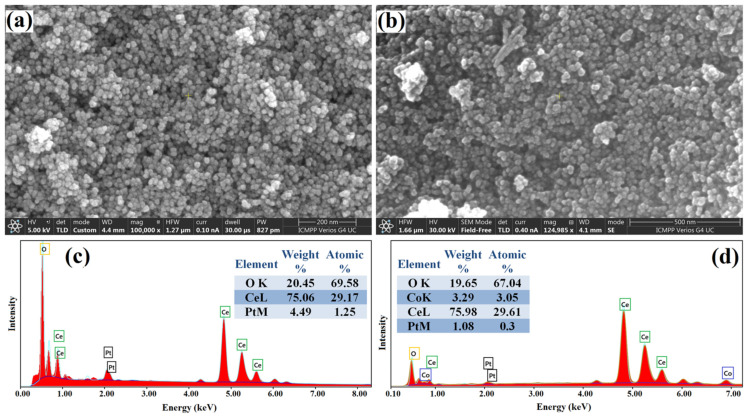
SEM images of V2 (**a**) and V2-Co (**b**) samples and EDX elemental analysis spectra of V2 (**c**) and V2-Co (**d**).

The morphology and elemental composition of Ceria and Co-doped CeO_2_ nanoparticles were investigated by SEM-EDX analysis; Figure 3a–d displays representative images for the V2 (Figure 3a,c) and V2-Co (Figure 3b,d) samples. As shown in Figure 3a,b, the prepared nanoparticles are characterized by a uniform spherical morphology that had not been significantly altered by doping with cobalt. To investigate the surface chemical composition of the materials, energy dispersive X-ray (EDX) analysis was performed. As shown in Figure 3c, on the surface of V2 sample, Ce and O elements (the peak for Pt being attributed to the sputter coating of the samples with a Pt layer) were identified. The EDX spectrum of the V2-Co sample (Figure 3d) confirms the existence of Co atoms in the CeO_2_ particles.

**Figure 4:**
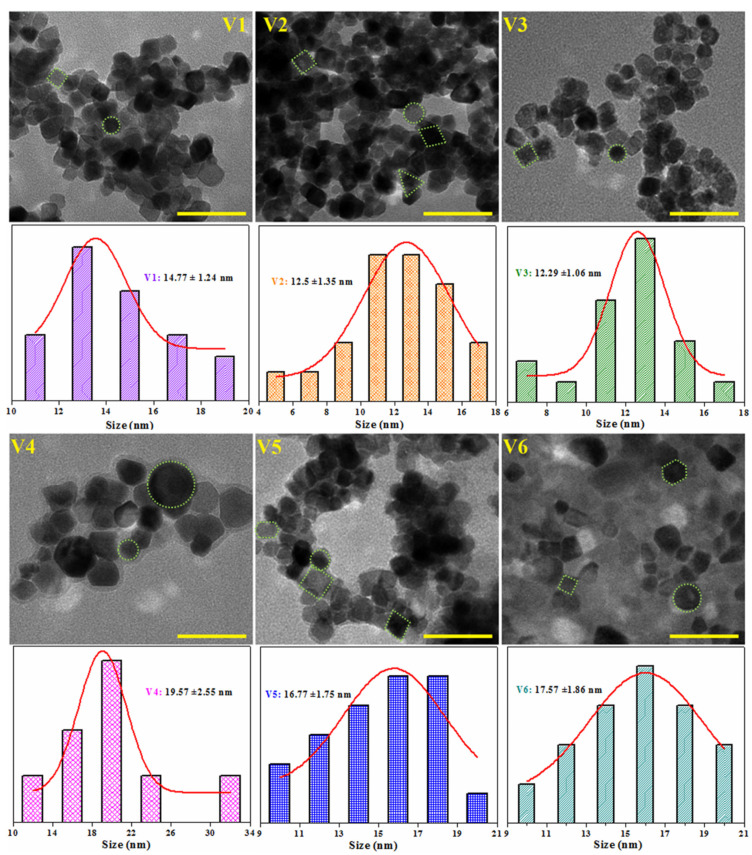
TEM images of CeO_2_ nanoparticles and the corresponding particle size distribution. Scale bar is 50 nm.

The morphology of the synthesized CeO_2_ NPs was also analyzed by TEM microscopy and the corresponding TEM images are shown in Figure 4.

As illustrated in the figure, in general, CeO_2_ nanoparticles have a narrow dimensional distribution, with crystal diameters between 12 and 20 nm (Table 2); however, a significant aggregation of individual units can be observed in the displayed microscopic images, which could be attributed to the deposition and drying process on copper holders [47]. All samples contain nearly spherical CeO_2_ nanoparticles, but cubic, rhomboidal, triangular, or polyhedral nanoparticles are also visible by TEM images. The largest inhomogeneous NPs are observed in the V4 sample (see the associated histogram in Figure 4), demonstrating that an increase in pH value is not favorable to the formation of nanoparticles with uniform size distribution. The estimated nanoparticle size resembles the crystallite size calculated from XRD data using Scherer’s formula (Table 2), sustaining once again that the V1–V3 samples (prepared at pH 8 and 10) are more dimensionally appropriate to be used as catalysts.

**Figure 5:**
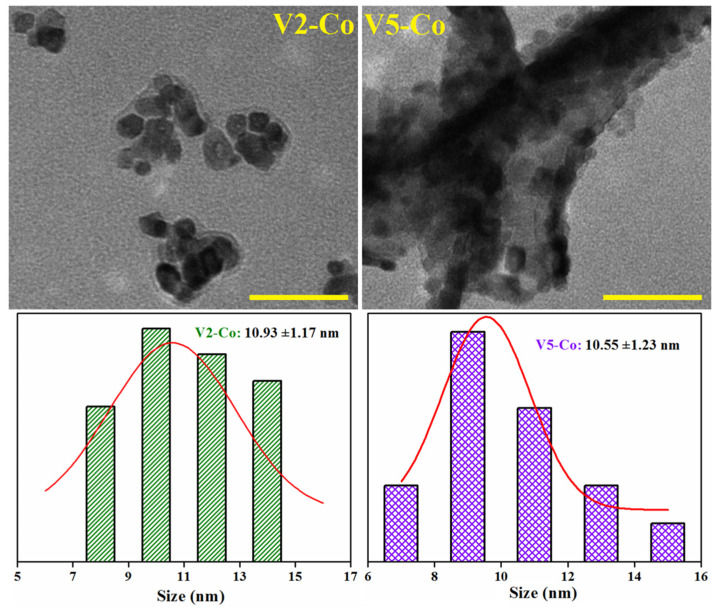
TEM images of Co-doped CeO_2_ nanoparticles and the corresponding particle size distribution. Scale bar is 50 nm.

After cobalt doping, the morphology of the V2-Co nanoparticles is modified, observing the formation of nanoparticles with uneven size distribution (between 7 and 15 nm) and irregular structure (Figure 5), suggesting that cobalt ions influence the nanoparticles’ formation, especially in terms of their shape. Regarding the V5-Co sample, the estimated nanoparticles’ diameters ranged from 6 to 16 nm, exhibiting a good dimensional and shape homogeneity. It is evident that the addition of cobalt dopant can obstruct the growth of CeO_2_ crystalline domains in both cases, indicating the appearance of some distortions at the atomic level caused by Co ions [34], as also remarked in the XRD analysis. This finding may represent a positive effect on the photocatalytic performance of the Co-doped nanoparticles.

### 3.2. Optical Study of Pristine and Co-Doped CeO_2_ NPs

**Figure 6:**
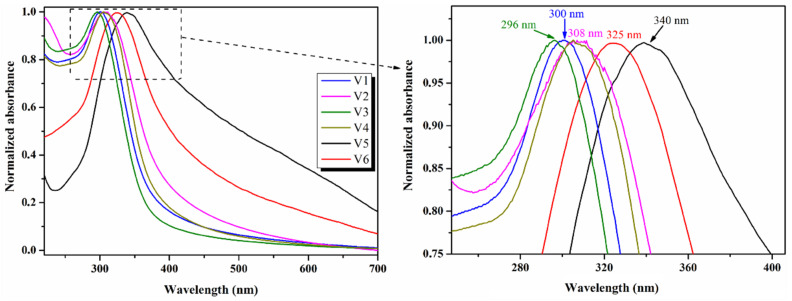
UV–Vis absorption spectra of pristine CeO_2_ NPs: V1–V6 samples.

Optical properties of the synthesized CeO_2_ NPs were studied with UV–Vis spectroscopy by measuring the absorbance and reflectance spectra of all samples at room temperature over a wavelength range of 200–1000 nm. Figure 6 presents the UV–Vis absorption spectra of pristine CeO_2_ NPs, namely V1–V6, and it can be observed that the prepared samples show a strong absorbance band in the UV range (296–340 nm). The absorption band in this region is mainly attributed to the charge transfer transition from O^2−^ in O *2p* to Ce^4+^ in Ce *4f* [48,49], but it is also commonly assigned to *4f*^1^*5d*^1^ states of the Ce^3+^ ion, commonly known as the f–f spin–orbit splitting of Ce *4f* states [50,51,52]. These findings are additional proof for the formation of CeO_2_ NPs with a cubic fluorite structure and the existence of Ce^3+^ ions in the structure [53] together with the oxygen vacancies formation.

The samples prepared at a basic pH (8, 10, and 12), namely V1–V4, have the absorption maxima in the UVB region of the spectrum (see Table 2). The absorption maximum of V1 nanoparticles (prepared at pH = 8, crystallite size ~12 nm) is at 300 nm, meanwhile the V2 and V4 samples (pH = 10 and 12, respectively) present a small red shift of the maxima at around 308 nm (Figure 6). The red shift of the V4 absorption band is attributed to the increase in the crystallite size of the nanoparticles (~22 nm), meanwhile V2, prepared at pH = 10, besides the absorption maximum red shift, also has a broader absorption band due to the mix of nanoparticles morphologies achieved (spheres, triangles, cubes, a.s.o.), as already presented in the TEM section. In the case of the V3 sample synthesized in same conditions as V2 (pH = 10), although having a longer reaction time (72 h), the obtained nanoparticles have more homogeneous shapes (predominant are nanoparticles with cubic-type morphology—Figure 4) and, consequently, the absorption band is sharper, and the maximum is blue shifted at 296 nm (Figure 6). Furthermore, it can be noticed that V5 and V6 nanoparticles prepared in an acidic medium (pH = 4) have higher values regarding the size of the crystallites (17.5–20 nm) and exhibit the absorption band maxima in the UVA region of the spectra (Table 2).

The absorption band of the V5 sample (3 h reaction time, CeO_2_ nanoparticles of different shapes) is very broad, with a maximum at *λ* = 340 nm. The increase in the reaction time to 72 h for the V6 sample leads to the formation of nanoparticles with cubic-type morphology, and this is reflected in the UV–Vis spectrum by the hypsochromic shift of the absorption band, together with its narrowing. Another interesting observation is that the adsorption band edges of the V5 and V6 samples are shifted to longer wavelengths in the visible domain, with V5 and V6 (pH = 4) presenting very broad absorption bands compared to the samples prepared at the basic pH (V1–V4).

**Figure 7:**
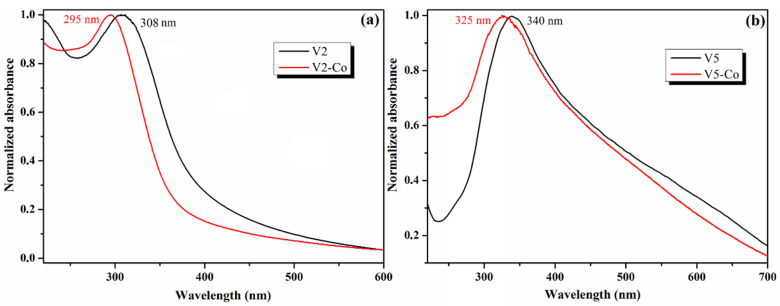
UV–Vis absorption spectra of V2 samples, pristine and Co-doped (**a**), and of V5 samples, pristine and Co-doped (**b**).

The influence of the Co-doping of CeO_2_ nanoparticles is reflected in the UV–Vis absorption properties, as can be observed from Figure 7. The absorption spectra of the Co-doped CeO_2_ nanoparticles, V2-Co and V5-Co, have the same curve profile but are shifted by almost 15 nm to shorter wavelengths compared to pristine CeO_2_ samples, V2 and V5; the band maximum of V2-Co is at *λ* = 295 nm and the one of V5-Co is at *λ* = 325 nm. The Co-doping of the CeO_2_ NPs thus modifies the absorption properties of the nanoparticles.

**Figure 8:**
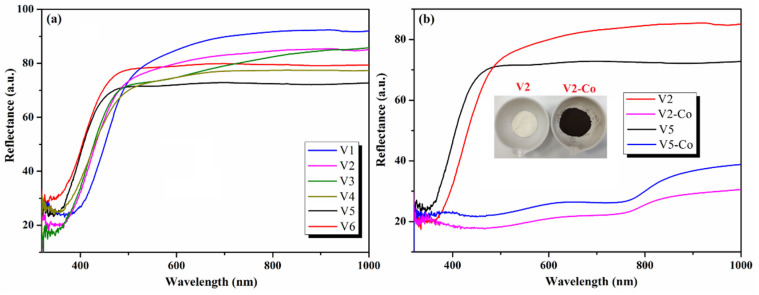
Reflectance spectra of pristine CeO_2_ NPs (V1–V6) (**a**) and of Co-doped CeO_2_ NPs (V2-Co, V5-Co) comparative to pristine V2 and V5 (**b**) (Inset: photo of V2 and V2-Co samples).

Moreover, the diffuse reflectance spectra of pristine and Co-doped CeO_2_nanoparticles (Figure 8) were registered in order to evaluate the band gap energy of our samples. As can be observed, the color of the pristine CeO_2_ NPs is pale-yellow, meanwhile Co-doped samples have a dark-brown color, and the chromic change is also reflected by DRS spectra, which show a decrease in reflectance (Figure 8b).

**Figure 9:**
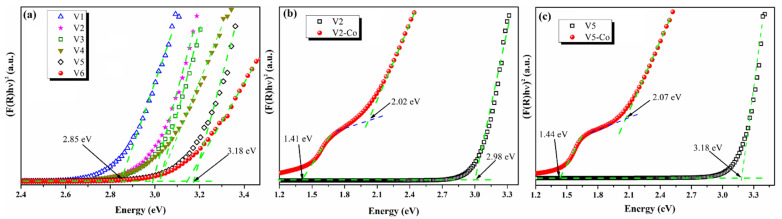
The Kubelka–Munk plots of different CeO_2_ NPs samples: V1–V6 (**a**), V2-Co comparative to V2 (**b**), and V5-Co relative to V5 (**c**).

The band gap energy indicates the threshold for photons to be absorbed and is mainly associated with the photocatalytic activity of the synthesized nanoparticles. The Kubelka–Munk theory in Equation (1) was used to determine the direct band gap values [48,54] of the pristine CeO_2_ NPs, V1–V6, and of the Co-doped CeO_2_ NPs, V2-Co and V5-Co.The corresponding plots are shown in Figure 9.

(h*υ* F(*R*))^2^ = A(h*υ* − *E_g_*) (1)

where F(*R*) = (1 − *R*)^2^/2*R*, h is a Planks constant, *υ* is the light frequency, A is the absorption coefficient and *E_g_* is the band gap energy.

The band gap energy values of the V1–V6 samples vary between 3.18 eV and 2.85 eV (Figure 9a) and, depending on the synthesis conditions used for the preparation of the nanoparticles, the calculated *E_g_* values are given in Table 2. V1–V4 samples, prepared in a basic medium, have lower *E_g_* values (2.85–3.02 eV) compared to the V5 and V6 samples (3.18 and 3.14 eV) synthesized in acidic conditions. In this regard, it has been reported that the low band gap energy values of the CeO_2_ nanoparticles are related to the abundance in oxygen vacancies and Ce^3+^ species in the nanostructure [55,56,57,58,59], which are furthermore linked to higher light absorption intensity, which, implicitly, could be reflected by the enhancement of the photocatalytic performance. According to these findings, V1–V4 samples synthesized at basic pH are recommended to be used as photocatalysts, since these CeO_2_ NPs will have higher photocatalytic performance compared to the ones prepared in an acidic medium (V5, V6).

V2, V3, and V4 nanostructures show similar band gap energy values (2.98 and 3.02 eV, respectively), meanwhile V1 has the lowest *E_g_* value, ~2.85 eV, suggesting that these type of CeO_2_ nanoparticles (achieved at pH = 8) can also be photoexcited by visible light irradiation. The response to the visible light of CeO_2_ NPs can lead to an enhancement of photocatalytic performance due to the generation of more electron-hole pairs [57], but they can also become potential candidates for photocatalyst under solar light irradiation. Hence, the V1 sample is more suitable for use in photocatalytic applications, rather than other synthesized pristine CeO_2_ NPs. We can conclude that, by choosing the optimal synthesis parameters, in this case the NH_4_OH co-reagent, pH = 8, *t* = 3 h, we can obtain pristine CeO_2_ NPs with desired values of *E_g_* and sizes for the envisaged application, namely photocatalysis under sunlight.

Furthermore, we investigate the effect of the Co-doping of the CeO_2_ nanoparticle on the band gap energy values of our samples, which were determined according to Makuła et al. [60]. We choose to use Co as the doping element since it has already been reported in the literature that CeO_2_ doping with Co could decrease the CeO_2_ band gap more than other transition metal dopants [61]. The Co-doping of V2 and V5 nanoparticles induces a significant decrease in the band gap energy values of the samples, as presented in Figure 9b,c. The *E_g_* of V2 decreases from 2.98 to 2.02 eV, meanwhile V5’s *E_g_* value is reduced from 3.18 to 2.07 eV by doping procedure. The Co-doping of CeO_2_ NPs leads to the release of oxygen ions, which favor the reduction inCe^4+^ to Ce^3+^; thus, the amount of Ce^3+^ states in the CeO_2_ structure increases and induces the formation of energy states located closer to the conduction band and reduces the band gap energy value [62,63]. The other direct band gap of about 1.4 eV identified in the Kubelka–Munk plots for V2-Co and V5-Co corresponds to the wurtzite CoO structure [64]. The narrowing of the band gap energy of CeO_2_ nanoparticles by Co-doping (~2.02–2.07 eV), along with the size reduction in the nanoparticles, transforms V2-Co and V5-Co samples in visible light-responsive materials and implicitly potential photocatalysts with high performance under sunlight.

**Figure 10:**
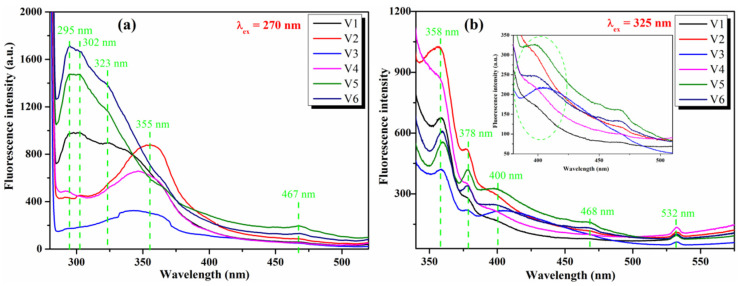
Fluorescence spectra of pristine CeO_2_ NPs (V1–V6) at *λ*_ex_ = 270 nm (**a**) and *λ*_ex_ = 325 nm (**b**).

Fluorescence spectroscopy offers us valuable information about the photogenerated electron-hole pairs at room temperature in semiconductor particles [52,65]. Generally, the intensity of fluorescence spectra is directly proportional to the ratio of the charge recombination. The fluorescence spectra of pure CeO_2_ NPs, V1–V6, were measured at two excitation wavelengths, namely at 270 nm and at 325 nm, as shown in Figure 10, and it can be observed that the emission curve profile of CeO_2_ NPs depends on the excitation wavelength used. At *λ*_ex_ = 270 nm (Figure 10a), V2–V4 samples, which have almost the same value of band gap energy (*E_g_* ~ 3.0 eV), displayed a broad emission band, with a maximum at 355 nm. V3 has a reduced fluorescence intensity comparative to V2 and V4, indicating that the recombination process of charge carriers is slower for V3 nanoparticles. V1, V5, and V6 nanoparticles have broader emission bands shifted to shorter wavelengths (~295–325 nm), with a reduced fluorescence intensity for V1.

The slow recombination rate of charge carriers for the V1 sample is an important advantage for the use of CeO_2_ NPs of this type as photocatalysts, together with their *E_g_* value (2.85 eV). At *λ*_ex_ = 325 nm (Figure 10b), all CeO_2_ samples (V1–V6) have similar emission curve profiles, with sharper bands with emission maxima positioned at: 358 nm, 378 nm, 400 nm, 468 nm, and 532 nm. A closer examination of the 400–500 nm wavelength area (Figure 10b—inset) revealed that emission intensity is lower for the V1 sample, sustaining the same conclusions as above (at *λ*_ex_ = 270 nm), according to which V1 nanoparticles present a slow recombination rate of charge carriers, a finding that is in concordance with XRD and TEM results; consequently, they will have improved photocatalytic activity.

**Figure 11:**
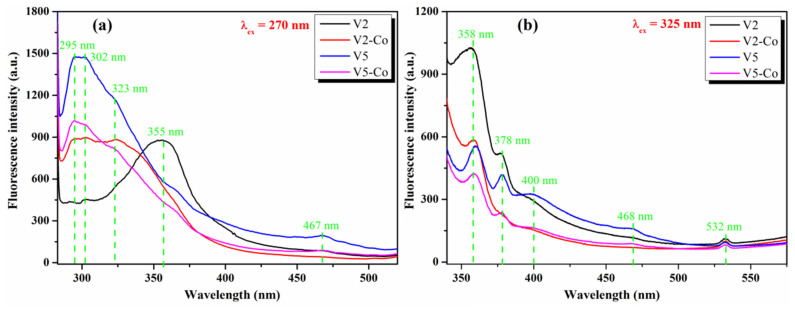
Fluorescence spectra of Co-doped CeO_2_ NPs (V2-Co, V5-Co) comparative to pristine V2 and V5 pristine CeO_2_ NPs at *λ*_ex_ = 270 nm (**a**) and *λ*_ex_ = 325 nm (**b**).

The fluorescence intensity of Co-doped CeO_2_ NPs, V2-Co and V5-Co, is lower than that of the pristine V2 and V5 at both excitation wavelengths *λ*_ex_ = 270 nm (Figure 11a) and *λ*_ex_ = 325 nm (Figure 11b). The reduction in fluorescence intensity by the Co-doping of CeO_2_ NPs shows that the charge recombination rate was diminished to the desired point in order to enhance the photocatalytic performance.

The XRD, TEM, and optical studies of all samples indicate that the best performance as a photocatalyst will be attained by V1 (pH = 8, *t* = 3h) and by the Co-doped samples, namely V2-Co and V5-Co. The photocatalytic behavior under visible radiation/sunlight of the three types of CeO_2_ NPs will be presented in detail in our future work, after their immobilization in a cellulose-based matrix, as previously described [48]. The nanoparticles’ immobilization will be a necessary step to confer reusability properties to the materials without having difficulties in their recovery.

## 4. Conclusions

The present study reports a preparation in various conditions (pH, time, co-reagent variation) of pristine CeO_2_ nanoparticles and also the preparation of some Co-doped CeO_2_ samples. All samples were characterized in detail (FTIR, XRD, SEM, EDX, TEM, UV–Vis, and fluorescence spectroscopy) to highlight the differences between them. The nanoparticles with smaller sizes (11–14 nm for V1–V3 samples) were achieved through the reaction realized in basic medium (pH = 8 and 10). The fluorescence investigation also showed that V1 has a slow recombination rate of charge carriers, and the UV–Vis studies revealed that the V1 sample has an *E_g_* value < 3.0 eV (~2.85 eV), meaning that these CeO_2_ NPs can be photoexcited by photons from a visible domain. Thus, in order to prepare pristine CeO_2_ NPs suitable for photocatalytic applications under sunlight, with desired values of *E_g_*, small sizes and slow recombination rate of charge carriers, the optimal synthesis parameters recommended are: NH_4_OH as co-reagent, reaction pH = 8, and reaction time *t* = 3 h. Moreover, the Co-doping of ceria nanoparticles is also recommended to prepare photocatalysts active under sunlight, since, through the Co-doping of CeO_2_ NPs (V2-Co and V5-Co), a decrease in the crystallite sizes/dimensions of the nanoparticles (10–12 nm) was achieved, together with the diminution of the charge recombination rate and of *E_g_* values (2.02–2.07 eV).

## Figures and Tables

**Table 1:** Experimental parameters for the preparation of undoped/doped CeO_2_ nanoparticles.

	Sample	Precursor	Co-Reagent	pH	Time (h)
1	V1	Ce(NO_3_)_3_·6H_2_O	NH_4_OH	8	3
2	V2	Ce(NO_3_)_3_·6H_2_O	NH_4_OH	10	3
3	V3	Ce(NO_3_)_3_·6H_2_O	NH_4_OH	10	72
4	V4	Ce(NO_3_)_3_·6H_2_O	NaOH	12	3
5	V5	Ce(NO_3_)_3_·6H_2_O	terephthalic acid	4	3
6	V6	Ce(NO_3_)_3_·6H_2_O	terephthalic acid	4	72
7	V2-Co	Ce(NO_3_)_3_·6H_2_O/Co(NO_3_)_2_·6H_2_O	NH_4_OH	10	3
8	V5-Co	Ce(NO_3_)_3_·6H_2_O/Co(NO_3_)_2_·6H_2_O	terephthalic acid	4	3

**Table 2:** Crystallite size (nm), average particle size (TEM, nm), and optical parameters (*λ*_abs_, *E_g_*) of pristine and Co-doped CeO_2_ NPs.

Sample	V1	V2	V3	V4	V5	V6	V2-Co	V5-Co
*d*_XRD_ (nm)	12.70	11.24	11.55	21.65	19.21	17.64	11.60	12.60
*d*_TEM_ (nm)	14.77	12.50	12.29	19.57	16.77	17.57	10.93	10.55
*λ*_abs_ (nm)	300	308	296	308	340	325	295	325
*E_g_* (eV)	2.85	2.98	3.02	3.02	3.18	3.14	2.02	2.07

## Data Availability

Data presented in this article are available at request from the corresponding author.
